# Molecular and Anti-Microbial Resistance (AMR) Profiling of Methicillin-Resistant *Staphylococcus aureus* (MRSA) from Hospital and Long-Term Care Facilities (LTCF) Environment

**DOI:** 10.3390/antibiotics10060748

**Published:** 2021-06-21

**Authors:** Bing-Mu Hsu, Jung-Sheng Chen, I-Ching Lin, Gwo-Jong Hsu, Suprokash Koner, Bashir Hussain, Shih-Wei Huang, Hsin-Chi Tsai

**Affiliations:** 1 Department of Earth and Environmental Sciences, National Chung Cheng University, Chiayi County 621, Taiwan; bmhsu@ccu.edu.tw (B.-M.H.); suprokashkoner@alum.ccu.edu.tw (S.K.); bashir@alum.ccu.edu.tw (B.H.); 2 Center for Innovative on Aging Society, National Chung Cheng University, Chiayi County 621, Taiwan; 3 Department of Medical Research, E-Da Hospital, Kaohsiung City 824, Taiwan; ed113187@edah.org.tw; 4 Department of Family Medicine, Asia University Hospital, Taichung City 413, Taiwan; D52282@auh.org.tw; 5 Department of Kinesiology, Health and Leisure, Chienkuo Technology University, Chenghua County 500, Taiwan; 6 Division of Infectious Diseases, Ditmanson Medical Foundation, Chia-Yi Christian Hospital, Chiayi City 600, Taiwan; 01347@cych.org.tw; 7 Department of Biomedical Sciences, National Chung Cheng University, Chiayi County 621, Taiwan; 8 Center for Environmental Toxin and Emerging Contaminant Research, Cheng Shiu University, Kaohsiung City 830, Taiwan; envhero@gcloud.csu.edu.tw; 9 Super Micro Research and Technology Center, Cheng Shiu University, Kaohsiung City 830, Taiwan; 10 Department of Psychiatry, School of Medicine, Tzu Chi University, Hualien County 970, Taiwan; 11 Department of Psychiatry, Tzu-Chi General Hospital, Hualien County 970, Taiwan

**Keywords:** methicillin-resistant *Staphylococcus aureus* (MRSA), long term care facilities (LTCF), multidrug resistance (MDR), *SCCmec* typing, enterobacterial repetitive intergenic consensus-polymerase chain reaction (ERIC-PCR)

## Abstract

To provide evidence of the cross-contamination of emerging pathogenic microbes in a local network between long-term care facilities (LTCFs) and hospitals, this study emphasizes the molecular typing, the prevalence of virulence genes, and the antibiotic resistance pattern of methicillin-resistant *Staphylococcus aureus*. MRSA isolates were characterized from 246 samples collected from LTCFs, medical tubes of LTCF residents, and hospital environments of two cities, Chiayi and Changhua. Species identification, molecular characterization, and drug resistance analysis were performed. Hospital environments had a higher MRSA detection rate than that of LTCF environments, where moist samples are a hotspot of MRSA habitats, including tube samples from LTCF residents. All MRSA isolates in this study carried the exfoliative toxin *eta* gene (100%). The majority of MRSA isolates were resistant to erythromycin (76.7%), gentamicin (60%), and ciprofloxacin (55%). The percentage of multidrug-resistant MRSA isolates was approximately 50%. The enterobacterial repetitive intergenic consensus polymerase chain reaction results showed that 18 MRSA isolates belonged to a specific cluster. This implied that genetically similar isolates were spread between hospitals and LTCFs in Changhua city. This study highlights the threat to the health of LTCFs’ residents posed by hospital contact with MRSA.

## 1. Introduction

In the past few decades, long-term care facilities (LTCFs) have faced an extreme threat from multidrug resistant (MDR) infectious pathogens [1,2]. The most abundant opportunistic pathogen is methicillin-resistant *Staphylococcus aureus* (MRSA), which not only affects healthcare centers, but is also associated with community and livestock infections [3,4,5]. The presence of *SCCmec* elements, consisting of *mecA* and *psm-mec* genes, enables resistance to various antibiotics including cephalosporins, oxacillin, imipenem, nafcillin, and p-lactam [6,7,8]. The most common molecular mechanism that enables resistance to these kinds of antibiotics is the *mecA* gene, which can synthesize PBP2A (a low-affinity penicillin-binding protein) and enables the continued synthesis of staphylococcal cell wall peptidoglycan through peptidoglycan transpeptidation when a high amount of beta-lactam antibiotics is present [9]. The *mecA* gene is situated on the staphylococcal cassette chromosome *mec* (*SCCmec*). Depending on the position of the *mec* and *ccr* complex sequences, *SCCmec* can be divided into I-to-VII types [10]. Globally, hospital-associated MRSA infections have been shown to be highly related to types I, II, and III of *SCCmec* and are sometimes enhanced by *SCCmec* IV. Moreover, community-associated MRSA infections commonly have a relationship with *SCCmec* type IV, V, or VII [10,11,12]. In Asia, there is strong evidence that the above-mentioned strains of MRSA generate ubiquitous epidemic nosocomial infections for health care and LTCF residents [13,14]. The previous study highlighted the presence of various virulence factors such as toxic shock syndrome toxin-1 (*tsst-1*), Panton–Valentine leukocidine (PVL), enterotoxins (*entA*-*E*), and exfoliative toxin (*eta*, *etb*) in MRSA isolates. These could enhance the severity of the infection during the pathogenesis process [15].

Continued spreading of infection-related diseases in hospitals and LTCF environments through these pathogens is not only hard to control but also requires proper surveillance, since they are not susceptible to antibiotic treatment [16,17,18]. Polymerase chain reaction (PCR)-based tools are commonly specific, low-cost methods to identify MDR and virulence genes, providing significant data during surveillance in hospital and LTCF environments [19,20]. For example, enterobacterial repetitive intergenic consensus sequence targeted PCR (ERIC-PCR) enables strain-level identification of these pathogens at the molecular level to categorize their genotyping in a range of evolutionary and epidemiological aspects [21]. Moreover, antibiotic susceptibility tests against largely populated antibiotics for these bacteria [22]. The distribution of these bacteria in hospital or LTCF environments has been well reported, although there is poor documentation of the pattern of distribution in terms of the infected hosts moving between these environments [23,24,25,26].

MRSA cross-contamination between hospitals and LTCFs is an important issue for public health, and most related research has focused on patients. In this study, our objectives were to classify molecular patterns and provide hotspot information on the environmental MRSA in a hospital and an affiliated LTCF. ERIC-PCR and *SCCmec* typing were performed for epidemiological typing of MRSA. Moreover, as the antibiotic resistance of MRSA is an urgent problem for public health, virulence gene profiling and antibiotic resistance were also conducted in this study.

## 2. Results

### 2.1. Detecting Rates of MRSA from LTCFs and Hospitals in Two Cities

A total of 246 LTCFs and hospital samples were collected to quantify the detection rate of MRSA. The overall percentage of MRSA prevalence in Changhua was 23.8% (39/164), and was 23.2% (19/82) in Chiayi (Table 1). The rate of MRSA in Changhua from LTCF (25%, 33/132) was higher than that from hospital samples (18.8%, 6/32). For Chiayi, samples from the hospital environment (25%, 8/32) contributed a higher rate than LTCF environment samples (22%, 11/50). For categorical division between arid versus moist samples, MRSA was detected in 22.2% (10/45) and 18.4% (7/38) of moist samples in the LTCF environments at Changhua and Chiayi, respectively; no MRSA was found in arid samples of LTCF from Changhua, although for Chiayi, 33.3% (4/12) were from arid samples. Thus, moist samples from Changhua and Chiayi hospitals accounted for 30% (*n =* 20) and 31.3% (*n =* 16) of the MRSA detection rate, respectively. Although the Changhua hospital arid samples had a detection rate of zero, this reached 18.3% (*n =* 16) for Chiayi hospital. The relative rate of MRSA detection included hospital environment samples for the outpatient floor (0%, *n =* 18, vs. 14.3% *n =* 7), inpatient floor (28.6%, *n =* 7, vs. 50%, *n =* 6), and used ward area (57.1%, *n =* 7, vs. 30%, *n =* 10) for Changhua versus Chiayi, respectively. In addition, the detection rate of MRSA in mild area samples was less frequent than that in the severe area in the LTCF environment of Chiayi (11.5%, *n =* 26; 33.3%, *n =* 24). In the Chiayi City hospital environment, the used ward samples had a higher prevalence rate of MRSA than the vacant ward samples (30%, *n =* 10 and 11.1%, *n =* 9, respectively). In addition, 63 indwelling medical tubes from LTCF residents of Changhua were tested for MRSA. The highest detection rate was obtained from nasogastric tubes, at 60% (*n =* 15), followed by tracheostomy tubes at 38.9% (*n =* 18), whereas the lowest detection rate was from Foley catheter-balloon samples (23.3%, *n =* 30).

### 2.2. SCCmec PCR Typing for Predicting the Source of the MRSA Strains

We purified 60 MRSA isolates from 246 samples. Their distribution in terms of sampling location and source, as well as different types of *SCCmec* PCR typing, are shown in Table 2. The majority of the 60 isolated MRSA strains belonged to *SCCmec* IV (48.3%, 29/60) and *SCCmec* III (41.7%, 25/60). Approximately 40% (24/60) of MRSA strains carried PVL toxin genes. In addition, 51.7% (31/60) of all strains were related to hospital-associated MRSA (HA-MRSA), 30% (18/60) were community-associated MRSA (CA-MRSA) and 18.3% (11/60) were livestock-associated MRSA (LA-MRSA). The *SCCmec* III group was detected in both hospital environments (50%) and LTCF samples (54%) in Changhua; however, in Chiayi, this group was present at a higher percentage in hospital samples (37.5%) compared with that found in LTCF samples (9.1%). For *SCCmec* IV detection, the percentage in LTCF samples was much higher than in hospital samples for both Changhua (40% vs. 16.7%, respectively) and Chiayi (90.9% vs. 50%, respectively).

For MRSA, 31.4% of LTCF samples in Changhua but none of the hospital samples contained the PVL gene, whereas in Chiayi, the percentage of LTCF samples with PVL (64.6%) was less than that of hospital samples (75%). The detection percentages of HA-MRSA, CA-MRSA, and LA-MRSA in Changhua were 63.4%, 19.5%, and 17.1% (*n =* 41), respectively; isolates from the hospital environment accounted for 83.3% (*n =* 6) of HA-MRSA, 0% of CA-MRSA (*n =* 6), and 16.7% of LA-MRSA (*n =* 6). Samples from LTCF resident tubes contained higher amounts of HA-MRSA strains (69.6%, *n =* 23, vs. 41.7%, *n =* 12, respectively) and CA-MRSA (26.1%, *n =* 23, vs. 16.7%, *n =* 12, respectively) than those from the LTCF environment, although isolates from LTCF environments were mostly related to LA-associated MRSA (41.7%, *n =* 12) than to isolates from tube samples of LTCF residents (4.3%, *n =* 23). For Chiayi-isolated MRSA strains, 26.3% (*n =* 19) were HA-MRSA, 52.6% were CA-MRSA, and 21.1% were related to LA-MRSA. Furthermore, 50% (*n =* 8) of MRSA prevalence was HA-MRSA from the hospital environment, although, for the LTCF environment, this amount was lower (9.1%, *n =* 11); however, the detection rates of CA- and LA-associated MRSA isolates in LTCF environment samples (63.3% and 27.3%; *n =* 11, respectively) were higher than those collected from the hospital environment (37.5% and 12.5%; *n =* 8, respectively).

### 2.3. The Detection of Toxin Genes by PCR

All isolated MRSA strains carried the exfoliative toxin *eta* gene (100%, 60/60) (Table 3). A lower number of enterotoxin genes were present, including *entA*, *entB*, and *entC* (3.3%, 16.7%, and 13.3%, respectively), but no samples carried the *entD*, *entE*, or *tsst-1* genes. In Changhua samples, the *entA* (16.7%, *n =* 6) and *eta* (100%, *n =* 6) genes were detected in isolated MRSA strains from the hospital environment, whereas other toxin genes were not found in all other isolates from this area. However, for MRSA strains from LTCF environment, the only genes detected were *entC* and *eta* (8.3% and 100%; *n =* 12, respectively). Enterotoxin genes *entB* (34.8%), *entC* (26.1%), and *eta* (100%) were found in MRSA isolates from medical tubes of LTCF residents. In Chiayi, isolates from the LTCF environment only carried the *eta* gene, whereas MRSA isolates from the hospital environment carried the *entA*, *entB*, *entC*, *etb*, and *eta* genes (12.5%, 25%, 12.5%, 25%, and 100%, *n =* 8, respectively).

### 2.4. Antimicrobial Susceptibility of the MRSA Strains

Eight types of antibiotics were used to analyze the antibiotic resistance capability of the 60 isolated MRSA strains. Overall, the resistance was ranked in order of highest to lowest as erythromycin, gentamicin, ciprofloxacin, clindamycin, tetracycline, sulfamethoxazole-trimethoprim, rifampicin, and chloramphenicol (Table 4). MRSA isolates from Changhua city had higher antimicrobial resistance compared to the Chiayi city. In addition, the Changhua hospital environment’s isolates had a higher percentage of resistance against most of the antibiotics, except chloramphenicol and rifampicin, compared to Changhua LTCF environment’s isolates. However, a higher percentage of isolates from Changhua LTCF resident tube samples were capable of resisting gentamicin. All the isolates from the Chiayi city LTCF environment were resistant to ciprofloxacin and erythromycin (100%, *n =* 11), and only one isolate was resistant to tetracycline and gentamicin. In the case of the Chiayi hospital environment, although the percentages of isolates able to resist the ciprofloxacin and erythromycin (50% and 62.5%, *n =* 8) were observed, this value was significantly higher in Chiayi LTCF environment samples.

For MDR profiling (Table 5), six MRSA isolates (83%) from the Changhua hospital environment had the highest degree of MDR. In the Changhua LTCF environment, the degree of MDR reached 50% (*n =* 12). In addition, for tube samples, 65.2% (*n =* 23) of isolates were found to be MDR. For Chiayi, only 37.5% (*n =* 8) of the total isolates from the hospital environment were MDR, whereas in the LTCF environment, only one isolate was MDR (9.1%).

### 2.5. Genetic Diversity Analysis by ERIC-PCR 

**Figure 1:**
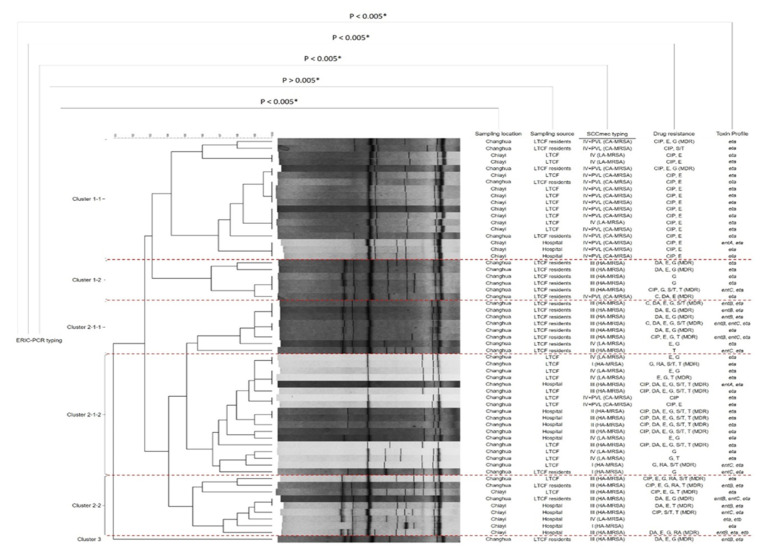
Genetical diversity of MRSA strains by ERIC-PCR combined with MDR pattern, *SCCmec* typing and toxin profile.

The combination of ERIC-PCR analysis with strain information for the 60 MRSA isolates is shown in Figure 1. The MRSA isolates in this study could be divided into three clusters by ERIC-PCR analysis: cluster 1 (24 isolates), cluster 2 (35 isolates), and cluster 3 (one isolate). We further divided cluster 1 into two sub-clusters (Cluster 1-1 and Cluster 1-2). Cluster 1-1 contained more MRSA strains than Cluster 1-2, and maximum number of this cluster’s isolates were isolated from Chiayi city samples predominantly consist of *SCCmec* type IV + PVL genes, classified as community associated, non-MDR MRSA clones, and only carried the *eta* toxin gene. The MRSA isolates that belonged to Cluster 1-2 (6 isolates) were related to HA-MRSA and contained *SCCmec* element III and the *eta* toxin gene. These isolates were MDR strains from Changhua city’s LTCF resident samples. Cluster 2 included three sub-clusters: 2-1-1, 2-1-2, and 2-2. The MRSA isolates of sub-cluster 2-1-1 presented a similar genetic profile as cluster 1-2, with the exception of the *entB* gene, which was present only in cluster 2-1-1. 

Furthermore, all isolates from clusters 2-1-1 and 1-2 were isolated from the dwelling medical tubes of Changhua LTFC residents. Eighteen MRSA isolates belonging to Cluster 2-1-2 showed high MDR properties combined with the *eta* gene and all were isolated from the hospital and LTCF environments of Changhua. The MRSA isolates of Cluster 2-2 were isolated from a range of samples, and the HA-MRSA isolates that contained *SCCmec* III elements and carried more than one toxin gene (*entB*, *entC*, *eta*, etc.) were predominant in this cluster. Cluster 3, considered as the outgroup, contained only one MRSA isolate isolated from a Changhua LTCF resident’s tube sample.

The chi-square test was used to evaluate the discriminatory power of ERIC-PCR associated with the factors of all isolates, for example, sampling location, sampling source, *SCCmec* typing, drug resistance, and toxin gene profile. This showed that only the sampling source (P > 0.05) had no significant association with ERIC-PCR analysis while all the remaining parameters had a significant association with cluster classification of ERIC-PCR.

## 3. Discussion

MRSA is one of the most predominant multidrug-resistant pathogens worldwide, and Asia is among the regions with the highest incidence in the world [27,28,29]. The estimated percentage of MRSA in hospital samples varies from 28% to >70% in Asia [30]. Studies regarding hospital environmental MRSA showed that the average prevalence was 2.2% and 11.8% in Ireland and Canada, respectively [31,32]. Several studies have focused on the isolation of MRSA from hospital curtains with MRSA detection percentages ranging from 15.5% to 31.6% [26,29,33]. In our case, MRSA prevalence in hospital environment samples was 18.8% in Changhua city and 25% in Chiayi city. The differences in MRSA prevalence in hospital and LTCF environments may be due to the sample type and local hygiene conditions. A UK study showed that MRSA detection rates were 40% and 17% in LTCF environmental samples in 2011 and 2013, respectively [34]. This study also implied that the lower frequency in 2013 might be due to improved infrastructure in the new LTCF. This agreed with our results that showed a lower MRSA detection percentage in Chiayi LTCF, which had a better hygiene environment compared to that of Changhua LTCF. Liu et al. demonstrated a disparity in MRSA detection rates between six LTCFs and concluded that improved infrastructure and a good hygiene environment constitute a powerful approach for reducing MRSA prevalence [27]. Our results implied that MRSA strains were present with high occurrence in moist environments, which agreed with other investigations, as moist samples act as prominent hotspots of MRSA distribution [7,8]. Here, the highest detection rate of MRSA was in nasogastric tubes, which are frequently used in LTCF residents.

The predominant *SCCmec* types were *SCCmec* III and IV, which agrees with studies of LTCFs in Taiwan [27,29]. However, the predominant *SCCmec* types in the two hospital environments of this study were different. The dominant type was *SCCmec* III in Chunghua hospital but was *SCCmec* IV in Chiayi hospital. HA-MRSA (31 isolates) was the dominant classification in this study, where HA-MRSA typically belongs to *SCCmec* I, II, and III [30]. Clinical studies in Taiwan also indicated that most of the HA-MRSA strains from hospitals belonged to *SCCmec* III [30,35,36]. Between 17.1% and 21.1% of LA-MRSA strains were isolated from hospitals and CTCFs, respectively, and all LA-MRSA isolated here belonged to *SCCmec* IV+PVL. This type is the same as our previous LA-MRSA study in the river basin of Chiayi [37]. Huang and Che’s study also concluded that more than 80% of Asian-specific LA-MRSA strains in Taiwan carried *SCCmec* IV and PVL genes, which strengthens our results [38].

The *eta* and *etb* enterotoxin-associated genes and tsst-1 are frequently prevalent virulence genes that vary between countries [30,33,39]. All the MRSA strains isolated in this study carry the eta gene; eta is reported to be the most privileged virulence factor-encoded gene, although this is contradicted in some reports [33,40,41,42]. Additionally, in contrast to other studies, we did not detect any *entD*, *entE*, and tsst-1-positive strains [33,42]. This showed that the distribution of toxin profiles of MRSA was highly diverse and implied geographic differences in these distributions. The MRSA isolates carrying the *entB* or *entC* genes all belonged to *SSCmec* III, except for one CA-MRSA isolate that carried two toxin genes, whereas all other isolates carried the eta gene only. This result is similar to the marginal differences identified in the distribution of toxin genes with *SCCmec* types reported by Fooladi et al. [33].

Antibiotic susceptibility testing revealed that MRSA isolates were more resistant to ciprofloxacin, erythromycin, and gentamicin than to other antibiotics, and 50% of these were related to MDR. MRSA from the hospital environment had more MDR ability compared with that from the LTCF environment. Furthermore, the prevalence of MDR MRSA was higher in Changhua city than in Chiayi city, and these were mainly HA-MRSA strains, followed by CA-MRSA from hospital environmental samples and LTCF resident samples, which agreed with a previous report [43]. Twenty-six of a total of 30 MDR MRSA isolates were identified in Changhua city and, among them, seven isolates were resistant to six antibiotics; this is an urgent and concerning issue and implies a cross-contamination problem.

ERIC-PCR is one of the most effective DNA-based molecular typing methods used to categorize MRSA isolates associated with epidemiological modeling and helps to track their spreading route [44]. In our ERIC-PCR fingerprinting, most of the MRSA isolates carrying the *SCCmec* IV + PVL genes (CA-MRSA) were from Chiayi LTCF environment samples and belonged to Cluster 1-1. Sub-clusters 1-1 and 2-1-1 were all from the Changhua LTFC resident samples, indicating that there were two main types of MRSA distributed in these LTCF residents.

In contrast, isolates in sub-cluster 2-1-1 from Changhua hospital and LTCFs generally consisted of *SCCmec* III (HA-MRSA) with MDR isolates. This finding implied that a genetically similar type of MRSA had spread between LTCFs and hospitals. ERIC-PCR aided the discrimination of resistance patterns among *SCCmec* types reported in our previous study [37]. ERIC-PCR is a popular tool for determining the genetic relatedness of different multidrug-resistant pathogenic bacteria from environmental samples, and previous analyses are consistent with our results [45,46]. This conclusion is further supported by the chi-square statistical hypothesis, where the individual significance score was < 0.05 with ERIC-PCR results. A similar analysis, adopted by Akindolire et. al. to distinguish the genetic diversity of isolated MRSA strains from their samples, strongly supported our ERIC-PCR results [47]. Therefore, using the ERIC-PCR figure combined with more detailed information, including toxin profiles, sampling location, other typing methods, and drug-resistant profiles, is a favorable approach to provide a complete understanding of pathogen transmission, microbial contamination, and surveillance spot information.

## 4. Materials and Methods

### 4.1. Sampling Information and Collection Method

Samples were taken from the hospital and LTCF environment of two cities in Taiwan (Changhua and Chiayi). For Changhua, the collected samples were from Hanming Christian Hospital (24.061271° N, 120.535643° E) and affiliated nursing homes (Auspicious Long-Term Care Center) (24.080745° N, 120.545247° E). Similarly, for Chiayi city, we collected samples from Chiayi Christian Hospital (23.499264° N, 120.450161° E) and affiliated nursing homes (Pau-Kang Long Term Care Center) (23.506819° N, 120.450287° E). A total of 246 samples were collected from LTCFs and hospital environments of both cities, including from medical tubes of LTCF residents, which are further categorized according to their type details, are described in Supplemental Table S1. Sterile cotton swabs were used to collect bacterial samples from the surface of each sampling point. Swabs were soaked in 5 mL of sterile phosphate buffer saline (PBS) in a centrifuge tube for storage and transported, at low temperature, to the laboratory for analysis.

### 4.2. Isolation Method of MRSA

To isolate MRSA from collected samples a two-step selective culture process was used. For the growth of MRSA colonies, we used CHROMagar™ MRSA (TPM ready-to-use media) and Baird-Parker agar (TPM ready-to-use media). The colonies that were grown on the respective cultured agar plate after the incubation period (30 °C for 24 h), were transferred into a sterile test tube containing brain heart infusion broth for pure culture [48]. Species identification, molecular characterization, and drug resistance analysis of isolated colony pure were performed using 300 µL broth from the enrichment culture.

### 4.3. PCR Identification of MRSA Strains

An aliquot of 300–600 μL of well-grown bacterial culture was centrifuged for 5 min at 10,000 rpm for DNA extraction. Genomic DNA was extracted using a commercial bacterial DNA extraction kit (MagPurix Bacterial DNA Extraction Kit, ZP02006, Taiwan, China). A reference bacterial genomic DNA (MRSA ATCC 29213) was used as a positive control (extracted by the same method and kit) [37]. After gDNA extraction, the PCR reaction mixture was prepared using 300 μg of gDNA with primers and master mix (Fast-RunTM Taq Master Mix with Dye). The total PCR reaction volume was 25 μL, and PCR reaction conditions are described in Supplemental Table S2. ERIC-PCR was used for PCR-based typing, and BioNumerics software was used to analyze kinship typing [37,49]. The *nuc* and *mecA* genes were used for the identification of MRSA strain types [50]. *SCCmec* and Panton–Valentine leukocidine (PVL) were used to confirm the classification of *mec* elements [51]. Some enterotoxins, toxic shock syndrome toxin-1 (*TSST-1*), and exfoliative toxins (ETs) genes were targeted for detection within the *S. aureus*. Finally, all PCR products were assessed by electrophoresis (110 V, 30 min, 1.5% agarose gel) to check the respective gene-specific amplicons.

### 4.4. Antibiotic Susceptibility Test

The following antibiotics—chloramphenicol (30 µg), ciprofloxacin (5 µg), clindamycin (2 µg), erythromycin (15 µg), gentamicin (10 µg), tetracycline (5 µg), rifampicin (30 µg) and sulfamethoxazole-trimethoprim (23.75/1.75 µg)—were used to test the antibiotic resistance of the MRSA isolates using the disc diffusion method as per the guidelines of the Clinical and Laboratory Standards Institute (CLSI, I-M45-P, 2006). These tests were conducted on Mueller–Hinton agar plates (TPM Ready-to-use media) [37].

## 5. Conclusions

This study showed that MRSA occurs at relatively higher rates in moist samples, especially in LTCF resident dwelling tubes. The eta gene was commonly found in all MRSA isolates. The hospital environmental MRSA isolates from Chiayi city carried the highest amount of enterotoxin genes, such as *entA*, *entB*, and *entC*, compared with those from Changhua city. Additionally, the MRSA isolates from the hospital environment and LTCF resident dwelling tubes exhibited high percentages of multidrug resistance to the following antibiotics: ciprofloxacin, clindamycin, erythromycin, sulfamethoxazole-trimethoprim, and tetracycline. The multidrug resistance problem of healthcare-associated MRSA is more severe in Changhua city than in Chiayi city. The *SCCmec* III containing HA-MRSA was identified as rank one and PVL + *SCCmec* IV element carrying CA-MRSA was rank two group of MRSA strain among 60 isolated *S. aureus* clones. ERIC-PCR was an effective tool for epidemiological characterization of MRSA isolates. The presence of genetically similar and multidrug-resistant MRSA in hospitals and their affiliated LTCFs is a threat to public health and needs to be closely monitored and controlled.

## Figures and Tables

**Table 1:** Prevalence of MRSA from various samples.

Sampling Locations	Sampling Sources	Sample Types	Number (%)
Changhua city	LTCF environment	Moist samples (*n =* 45)	10 (22.2%)
		Arid samples (*n =* 24)	0 (0%)
		Total LTCF environment (*n =* 69)	10 (14.5%)
	LTCF dwelling medical tubes	Foley catheter-balloons from LTCF residents (*n =* 30)	7 (23.3%)
		Nasogastric tubes from LTCF residents (*n =* 15)	9 (60%)
		Tracheostomy tubes from LTCF residents (*n =* 18)	7 (38.9%)
		Total tubes from LTCF residents (*n =* 63)	23 (36.5%)
	Total LTCF samples (*n =* 132)		33 (25%)
	Hospital environment	Moist samples (*n =* 20)	6 (30%)
		Arid samples (*n =* 12)	0 (0)%
		Outpatient floor (*n =* 18)	0 (0%)
		Inpatient floor (*n =* 7)	2 (28.6%)
		Ward (used) (*n =* 7)	4 (57.1%)
		Total hospital samples (*n =* 32)	6 (18.8%)
	Total Changhua city samples (*n =* 164)		39 (23.8%)
Chiayi city	LTCF environment	Mild area (*n =* 26)	3 (11.5%)
		Severe area (*n =* 24)	8 (33.3%)
		Moist samples (*n =* 38)	7 (18.4%)
		Arid samples (*n =* 12)	4 (33.3%)
		Total LTCF samples (*n =* 50)	11 (22%)
	Hospital environment	Outpatient building 1F (*n =* 7)	1 (14.3%)
		Inpatient building 1F (*n =* 6)	3 (50%)
		Total building 1F (*n =* 13)	4 (30.8%)
		Ward (vacancy) (*n =* 9)	1 (11.1%)
		Ward (used) (*n =* 10)	3 (30%)
		Total wards (*n =* 19)	4 (21.1%)
		Moist samples (*n =* 16)	5 (31.3%)
		Arid samples (*n =* 16)	3 (18.8%)
		Total hospital samples (*n =* 32)	8 (25%)
	Total Chiayi city samples (*n =* 82)		19 (23.2%)
Total Changhua city and Chiayi city samples (*n =* 246)			58 (23.6%)

**Table 2:** *SCCmec* PCR typing results of the 60 MRSA isolates.

Sampling Locations	Sampling Sources	*SCCmec* I	*SCCmec* II	*SCCmec* III	*SCCmec* IV	*SCCmec* V	PVL	HA-MRSA (I, II, III)	CA-MRSA (IV+PVL, V+PVL)	LA-MRSA (IV, V)
Changhua City	LTCF environment (*n =* 12)	2 (16.7%)	0 (0%)	3 (25%)	7 (58.3%)	0 (0%)	2 (16.7%)	5 (41.7%)	2 (16.7%)	5 (41.7%)
	Tubes from LTCF residents (*n =* 23)	1 (4.3%)	0 (0%)	15 (65.2%)	7 (30.4%)	0 (0%)	9 (39.1%)	16 (69.6%)	6 (26.1%)	1 (4.3%)
	Total LTCF isolates (*n =* 35)	3 (8.6%)	0 (0%)	18 (51.4%)	14 (40%)	0 (0%)	11 (31.4%)	21 (60%)	8 (22.9%)	6 (17.1%)
	Hospital environment (*n =* 6)	0 (0%)	2 (33.3%)	3 (50%)	1 (16.7%)	0 (0%)	0 (0%)	5 (83.3%)	0 (0%)	1 (16.7%)
	Total Changhua city isolates (*n =* 41)	3 (7.3%)	2 (4.9%)	21 (51.2%)	15 (36.6%)	0 (0%)	11 (26.8%)	26 (63.4%)	8 (19.5%)	7 (17.1%)
Chiayi City	LTCF environment (*n =* 11)	0 (0%)	0 (0%)	1 (9.1%)	10 (90.9%)	0 (0%)	7 (63.6%)	1 (9.1%)	7 (63.6%)	3 (27.3%)
	Hospital environment (*n =* 8)	1 (12.5%)	0 (0%)	3 (37.5%)	4 (50%)	0 (0%)	6 (75%)	4 (50%)	3 (37.5%)	1 (12.5%)
	Total Chiayi city isolates (*n =* 19)	1 (5.3%)	0 (0%)	4 (21.1%)	14 (73.7%)	0 (0%)	13 (68.4%)	5 (26.3%)	10 (52.6%)	4 (21.1%)
Total Changhua City and Chiayi city isolates (*n =* 60)		4 (6.7%)	2 (3.3%)	25 (41.7%)	29 (48.3%)	0 (0%)	24 (40%)	31 (51.7%)	18 (30%)	11 (18.3%)

**Table 3:** PCR detection of toxin genes from the 60 MRSA strains.

Sampling Locations	Sampling Sources	*entA*	*entB*	*entC*	*entD*	*entE*	*Eta*	*etb*	*tsst-1*
Changhua city	LTCF environment (*n =* 12)	0 (0%)	0 (0%)	1 (8.3%)	0 (0%)	0 (0%)	12 (100%)	0 (0%)	0 (0%)
	Tubes from LTCF residents (*n =* 23)	0 (0%)	8 (34.8%)	6 (26.1%)	0 (0%)	0 (0%)	23 (100%)	0 (0%)	0 (0%)
	Total LTCF isolates (*n =* 35)	0 (0%)	8 (22.9%)	7 (20%)	0 (0%)	0 (0%)	35 (100%)	0 (0%)	0 (0%)
	Hospital environment (*n =* 6)	1 (16.7%)	0 (0%)	0 (0%)	0 (0%)	0 (0%)	6 (100%)	0 (0%)	0 (0%)
	Total Changhua City isolates (*n =* 41)	1 (2.4%)	8 (19.5%)	7 (17.1%)	0 (0%)	0 (0%)	41 (100%)	0 (0%)	0 (0%)
Chiayi city	LTCF environment (*n =* 11)	0 (0%)	0 (0%)	0 (0%)	0 (0%)	0 (0%)	11 (100%)	0 (0%)	0 (0%)
	Hospital environment (*n =* 8)	1 (12.5%)	2 (25%)	1 (12.5%)	0 (0%)	0 (0%)	8 (100%)	2 (25%)	0 (0%)
	Total Chiayi City isolates (*n =* 19)	1 (5.3%)	2 (10.5%)	1 (5.3%)	0 (0%)	0 (0%)	19 (100%)	2 (10.5%)	0 (0%)
Total Changhua city and Chiayi city isolates (*n =* 60)		2 (3.3%)	10 (16.7%)	8 (13.3%)	0 (0%)	0 (0%)	60 (100%)	2 (3.3%)	0 (0%)

**Table 4:** Antimicrobial susceptibility results of the 60 MRSA strains.

Sampling Locations	Sampling Sources	C	CIP	DA	E	G	RA	S/T	T	MDR
Changhua city	LTCF environment (*n =* 12)	0 (0%)	5 (41.7%)	2 (16.7%)	7 (58.3%)	10 (83.3%)	3 (25%)	5 (41.7%)	5 (41.7%)	6 (50%)
	tubes from LTCF residents (*n =* 23)	3 (13.3%)	8 (34.8%)	10 (43.5%)	17 (73.9%)	18 (78.3%)	1 (4.3%)	4 (17.4%)	4 (17.4%)	15 (65.2%)
	LTCF isolates (*n =* 35)	3 (8.6%)	13 (37.1%)	12 (34.3%)	24 (68.6%)	28 (80%)	4 (11.4%)	9 (25.7%)	9 (25.7%)	21 (60%)
	Hospital environment (*n =* 6)	0 (0%)	5 (83.3%)	5 (83.3%)	6 (100%)	6 (100%)	0 (0%)	5 (83.3%)	5 (83.3%)	5 (83.3%)
	Total Changhua city isolates (*n =* 41)	3 (7.3%)	18 (43.9%)	17 (41.5%)	30 (73.2%)	34 (82.9%)	4 (9.8%)	14 (34.1%)	14 (34.1%)	26 (63.4%)
Chiayi city	LTCF environment (*n =* 11)	0 (0%)	11 (100%)	0 (0%)	11 (100%)	1 (9.1%)	0 (0%)	0 (0%)	1 (9.1%)	1 (9.1%)
	Hospital environment (*n =* 8)	0 (0%)	4 (50%)	2 (25%)	5 (62.5%)	1 (12.5%)	1 (12.5%)	1 (12.5%)	2 (25%)	3 (37.5%)
	Total Chiayi city isolates (*n =* 19)	0 (0%)	15 (78.9%)	2 (10.5%)	16 (84.2%)	2 (10.5%)	1 (5.3%)	1 (5.3%)	3 (15.8%)	4 (21.1%)
Total Changhua city and Chiayi city isolates (*n =* 60)		3 (5%)	33 (55%)	19 (31.7%)	46 (76.7%)	36 (60%)	5 (8.3%)	15 (25%)	17 (28.3%)	30 (50%)

C: chloramphenicol; CIP: ciprofloxacin; DA: clindamycin; E: erythromycin; G: gentamicin; RA: rifampicin; S/T: sulfamethoxazole-trimethoprim; T: tetracycline; MDR: multidrug resistance.

**Table 5:** MDR pattern profile results of 30 MRSA strains.

Sampling Locations	Changhua City			Chiayi City		Total MDR Strains
Sampling Sources	LTCF Environment	Tubes from LTCF Residents	Hospital Environment	LTCF Environment	Hospital Environment	
CIP-DA-E-G-S/T-T (6 drugs)	2		5			7
C-DA-E-G-S/T (5 drugs)		2				4
CIP-E-G-RA-S/T (5 drugs)	1					
CIP-E-G-RA-T (5 drugs)		1				
CIP-E-G-S/T (4 drugs)				1		5
CIP-E-G-T (4 drugs)		1				
CIP-G-S/T-T (4 drugs)		1				
DA-E-G-RA (4 drugs)					1	
G-RA-S/T-T (4 drugs)	1					
C-DA-E (3 drugs)		1				14
CIP-E-G (3 drugs)		2				
CIP-ST-T (3 drugs)					1	
DA-E-G (3 drugs)		7				
DA-E-T (3 drugs)					1	
E-G-T (3 drugs)	1					
G-RA-S/T (3 drugs)	1					
Total strains	6	15	5	1	3	30

C: chloramphenicol; CIP: ciprofloxacin; DA: clindamycin; E: erythromycin; G: gentamicin; RA: rifampicin; S/T: sulfamethoxazole-trimethoprim; T: tetracycline; MDR: multidrug resistance.

## Data Availability

The data presented in this study are available on request from the corresponding author.

## Supplementary Materials

The following are available online at https://www.mdpi.com/article/10.3390/antibiotics10060748/s1, Table S1: Details of samples location, source, types and number, Table S2: MRSA strain identification (ERIC-PCR), *SCCmec* typing and enterotoxin gene detection condition of PCR with primers information.
