# Supplementary material for: Molecular and Anti-Microbial Resistance (AMR) Profiling of Methicillin-Resistant Staphylococcus aureus (MRSA) from Hospital and Long-Term Care Facilities (LTCF) Environment
Source: Antibiotics (Basel). 2021 Jun 21;10(6):748. doi: 10.3390/antibiotics10060748 (PMC8235027; doi:10.3390/antibiotics10060748)
Supplement: Supplementary file 1 [file antibiotics-10-00748-s001.zip › antibiotics-1235530-supplementary.pdf]

antibiotics

MDPI

Supplementary

Molecular and Anti-Microbial Resistance (AMR) Profiling of Methicillin-Resistant *Staphylococcus aureus* (MRSA) from Hospital and Long-Term Care Facilities (LTCF) Environment

Bing-Mu Hsu [1,11,†], Jung-Sheng Chen [2], I-Ching Lin [3,4,†], Gwo-Jong Hsu [5,†], Suprokash Koner [1,6,†], Bashir Hussain [1,6], Shih-Wei Huang [7,8] and Hsin-Chi Tsai [9,10,*]

Table **S1.** Details of samples location, source, types and number.

Sampling Location	Sampling source	Sample types	Number of samples	Total Samples
Changhua city	LTCF environment	Moist samples	45	69
		Arid sample	24	
	LTCF residents	Foley catheter-balloons from LTCF residents	30	63
		Nasogastric tubes from LTCF residents	15	
		Tracheostomy tubes from LTCF residents	18	
	Hospital environment	Outpatient floor	18	32
		Inpatient floor	7	
		Ward (used)	7	
	Changhua city's total samples			164
Chiayi city	LTCF environment	Mild area	26	50
		Severe area	24	
	Hospital environment	Outpatient building 1F	7	32
		Inpatient building 1F	6	
		Ward (vacant)	9	
		Ward (used)	10	
	Total Chiayi city samples			82
Total Changhua city & Chiayi city samples				246

Table S2. MRSA strain identification (ERIC- PCR), *SCCmec* typing and enterotoxin gene detection condition of PCR with primers information.

Target gene	Size	Sequence (5' to 3')	Reaction Materials (Final Volume: 25 µl)	PCR Condition	References
nuc<br>*mec*A	270<br>448	nuc-F 5′-GCGATTGATGGTGATACGGTT-3′<br>nuc-R 5′-AGCCAAGCCTTGACGAACTAAAGC-3′<br>mecA-F 5′-CTCAGGTACTGCTATCCACC-3′<br>mecA-R 5′-CACTTGGTATATCTTCACC-3′	DNA: 100-300 ng<br>Primer: 400 nM nuc FR & mecA FR<br>Master mix: 5 µl	Pre-denaturation: 95°C 5 min<br>Denaturation: 94°C 60s<br>Annealing: 55°C 60s<br>Extension: 72°C 60s<br>D.A.E. Cycles: 30 cycles<br>Final extension: 72°C 10 min	[1,2]
ERIC	----	ERIC1R: 5′-ATGTAAGCTCCTGGGGATTCAC-3′<br>ERIC2: 5′-AAGTAAGTGACTGGGGTGAGCG-3′	DNA: 100-300 ng<br>Primer: 500 nM ERIC1R & ERIC2<br>Master mix: 5 µ	Denaturation-1: 95°C 5 min<br>Annealing-1: 36°C 1 min<br>Extension-1: 72°C 4 min<br>D.A.E.-1 Cycles: 1 cycle<br>Denaturation-2: 95°C 1 min<br>Annealing-2: 36°C 1 min<br>Extension-2: 72°C 4 min<br>D.A.E.-2 Cycles: 35 cycles<br>Final extension: 72°C 8 min	[3]
SCCmec I<br>*SCCmec* II<br>*SCCmec* II, III<br>*SCCmec* III<br>*SCCmec* III<br>*SCCmec* I, II, IV	495<br>284<br>209<br>243<br>414<br>342	CIF2 F2: 5'-TTCGAGTTGCTGATGAAGAAGG-3'<br>CIF2 R2: 5'-ATTTACCACAAGGACTACCAGC-3'<br>KDP F1: 5'-AATCATCTGCCATTGGTGATGC-3'<br>KDP R1: 5'-CGAATGAAGTGAAAGAAAGTGG-3'	DNA: 100-300 ng<br>Primer:<br>400 nM CIF-FR,<br>200 nM KDP-FR,<br>200 nM RIFF3R9<br>400 nM MECI-FR,<br>400 nM RIFF10R13,<br>800 nM DCS-FR<br>Master mix: 5 µl	Pre-denaturation: 94°C 4 min<br>Denaturation: 94°C 30s<br>Annealing: 53°C 30s<br>Extension: 72°C 1 min<br>D.A.E. Cycles: 30 cycles<br>Final extension: 72°C 4 min	[4]

		MECI P2: 5’-ATCAA-GACTT-GCATTCAGGC-3’ MECI P3: 5’-GCGGTTTCAATTCACTT-GTC-3’ RIF F3: 5’-GTGATTGTTCGAGA-TATGTGG-3’ RIF R9: 5’-CGCTTTATCTGTATCTATCGC-3’ RIF F10: 5’-TTCTTAAGTACAC-GCTGAATCG-3’ RIF R13: 5’-GTCACAG-TAATTCCATCAATGC-3’ DCS F2: 5’-CATCC-TATGA-TAGCTT-GGTC-3’ DCS R1: 5’-CTAAATCATAGCCATGACCG-3’			
SCCmec V	325	Type V-F: 5’-GAACATT-GTTACTTAAATGAGCG-3' Type V-R: 5’-TGAAAGTT-GTACCCTT-GACACC-3'	DNA: 100-300 ng Primer: 100 nM V-FR Master mix: 5 µl	Pre-denaturation: 94°C 5 min Denaturation-1: 94°C 45s Annealing-1: 65°C 45s Extension-1: 72°C 1.5 min D.A.E.-1 Cycles: 10 cycles Denaturation-2: 94°C 45s Annealing-2: 55°C 45s Extension-2: 72°C 1.5 min D.A.E.-2 Cycles: 25 cycles Final extension: 72°C 10 min	[5]
PVL	433	PVL-1: 5’-ATCATT-AGGTAAAAT	DNA:100-300 ng Primer: 400 nM FR	Pre-denaturation: 94°C 5 min	[6]

		GTCTG-GACATGATCCA-3' PVL-2: 5'-GCATCAAGTGTATTGGA-TAGCAAAAGC-3'	Master mix: 5 µl	Denaturation: 94°C 40s Annealing: 53°C 40s Extension: 72°C 1 min D.A.E. Cycles: 35 cycles Final extension: 72°C 10 min	
*ent*A<br>*ent*B<br>*ent*C<br>*ent*D<br>*ent*E<br>tsst-1<br>*eta*<br>*etb*	121<br>478<br>459<br>384<br>495<br>271<br>464<br>200	entA-F: 5′-TTGGAAC-GGTTAAAACGAA-3′ entA-R: 5′-GAAC-CTTCCCATCAAAAACA-3′ entB-F: 5′-TCG-CATCAAACTGACAAACG-3′ entB-R: 5′-GCAGGTACTCTATAAGTGCC-3′ entC-F: 5′-GGAGGAA-TAACAAAACATGAAGG-3′ entC-R: 5′-AAAGGCAA-GCAC-CGAAGTAC-3′ entD-F: 5′-TGGTGGTGAAATAGA-TAGGAC-3′ entD-R: 5′-TGAAGGTGCTCTGTGGA-TAAT-3′ entE-F: 5′-TGG-TAGCGA-GAAAA-GCGAAG-3′ entE-R: 5′-TGTAAA-TAATGCCTT-GCCTGAA-3′ tsst-1-F: 5′-CTGG-TATAGTAG-TGGGTCTG-3′	DNA: 100-300 ng Primer: 400 nM Primer FR Master mix: 5 µl	Pre-denatura-tion: 94°C 5 min Denaturation: 94°C 1 min Annealing: 2 min Extension: 72°C 1 min D.A.E. Cycles: 35 cycles Final extension: 72°C 5 min Annealing-Temp. entA: 50°C entB: 55°C entC: 59°C entD: 51°C entE: 55.5°C tsst-1: 54°C eta: 54°C etb: 50.9°C	[7]

tsst-1-R: 5′-AG-<br>GTAGTTC-<br>TATTGGAG-<br>TAGG-3′<br>eta-F: 5′-TTT-<br>GCTTTCTT-<br>GATTT-<br>GGATTC-3′<br>eta-R: 5′-<br>GATGTGTTC<br>GGTTT-<br>GATTGAC-3′<br>etb-F: 5′-AC-<br>GGCTA-<br>TATACATTC<br>AATT-3′<br>etb-R: 5′-<br>TCCATCGA-<br>TAATATAC-<br>CTAA-3′

References

1. Brakstad, O.G.; Aasbakk, K.; Maeland, J.A. Detection of Staphylococcus aureus by polymerase chain reaction amplification of the nuc gene. *Journal of clinical microbiology* **1992**, *30*, 1654-1660.
2. Sakoulas, G.; Gold, H.S.; Venkataraman, L.; DeGirolami, P.C.; Eliopoulos, G.M.; Qian, Q. Methicillin-resistant Staphylococcus aureus: comparison of susceptibility testing methods and analysis of mecA-positive susceptible strains. *Journal of clinical microbiology* **2001**, *39*, 3946-3951.
3. Ye, Y.; Jiang, Y.; Fan, T.; Jiang, Q.; Cheng, Y.; Lu, J.; Lin, L. Resistance characterization, virulence factors, and ERIC-PCR fingerprinting of Aeromonas veronii strains isolated from diseased Trionyx sinensis. *Foodborne pathogens and disease* **2012**, *9*, 1053-1055.
4. Oliveira, D.C.; Lencastre, H.d. Multiplex PCR strategy for rapid identification of structural types and variants of the mec element in methicillin-resistant Staphylococcus aureus. *Antimicrobial agents and chemotherapy* **2002**, *46*, 2155-2161.
5. Zhang, K.; McClure, J.-A.; Elsayed, S.; Louie, T.; Conly, J.M. Novel multiplex PCR assay for characterization and concomitant subtyping of staphylococcal cassette chromosome mec types I to V in methicillin-resistant Staphylococcus aureus. *Journal of clinical microbiology* **2005**, *43*, 5026-5033.
6. Asghar, A.H. Molecular characterization of methicillin-resistant Staphylococcus aureus isolated from tertiary care hospitals. *Pakistan journal of medical sciences* **2014**, *30*, 698.
7. Fooladi, A.A.I.; Ashrafi, E.; Tazandareh, S.G.; Koosha, R.Z.; Rad, H.S.; Amin, M.; Soori, M.; Larki, R.A.; Choopani, A.; Hosseini, H.M. The distribution of pathogenic and toxigenic genes among MRSA and MSSA clinical isolates. *Microb Pathogenesis* **2015**, *81*, 60-66.
